# Acute ischemic stroke in low-voltage electrical injury: A case report

**DOI:** 10.4103/2152-7806.74093

**Published:** 2010-12-17

**Authors:** Yeh Huan-Jui, Liu Chih-Yang, Lo Huei-Yu, Chen Po-Chih

**Affiliations:** Department of Physical Medicine and Rehabilitation, Taoyuan General Hospital Department of Health, Taoyuan, Taiwan; 1 Department of Neurology, Buddhist Tzu Chi General Hospital, Taipei, Taiwan; 2 Department of Neurology, Taipei Medical University-Shung Ho Hospital, Taipei, Taiwan

**Keywords:** electrical injury, stroke, vessel spasm

## Abstract

**Background:**

Acute stroke is not a common complication of electrical injury, and only a few cases of acute stroke have been reported for lightning or high-voltage injuries.

**Case Report:**

We present the case of a man who suffered from a low-voltage electrical injury followed by ischemic stroke. Magnetic resonance angiography showed segmental narrowing of the right internal carotid artery and right middle cerebral artery. The patient underwent thrombolytic therapy and catheter-assisted angioplasty. The low-voltage current-induced vasospasm rather than direct vascular injury, and this may explain why the intracranial defect occurred away from the electrical current pathway.

**Conclusion:**

Electric shock injury with low-voltage alternating currents and prolonged contact period may cause ischemic stroke.

## INTRODUCTION

Electrical injuries are relatively common in daily life, and they are accidentally incurred. The severity of electrical injury varies depending on the magnitude of energy delivered, type of current, current pathway and duration of contact. Many organs or tissues, including the heart, muscles, kidneys, skin, and vascular and nervous systems are especially vulnerable to such injury. Acute stroke is a rare complication, though cases of acute stroke due to electrical injury have been reported when the voltage has been high, e.g., with lightning,[1] and electrical shock with voltages between 800 and 1500 V.[10]

Here, we describe the case of a man who suffered from an ischemic stroke after an accidental electrical injury resulting from a low-voltage alternating current. Such a case has not been reported before, to the best of our knowledge.

## CASE REPORT

**Figure 1:**
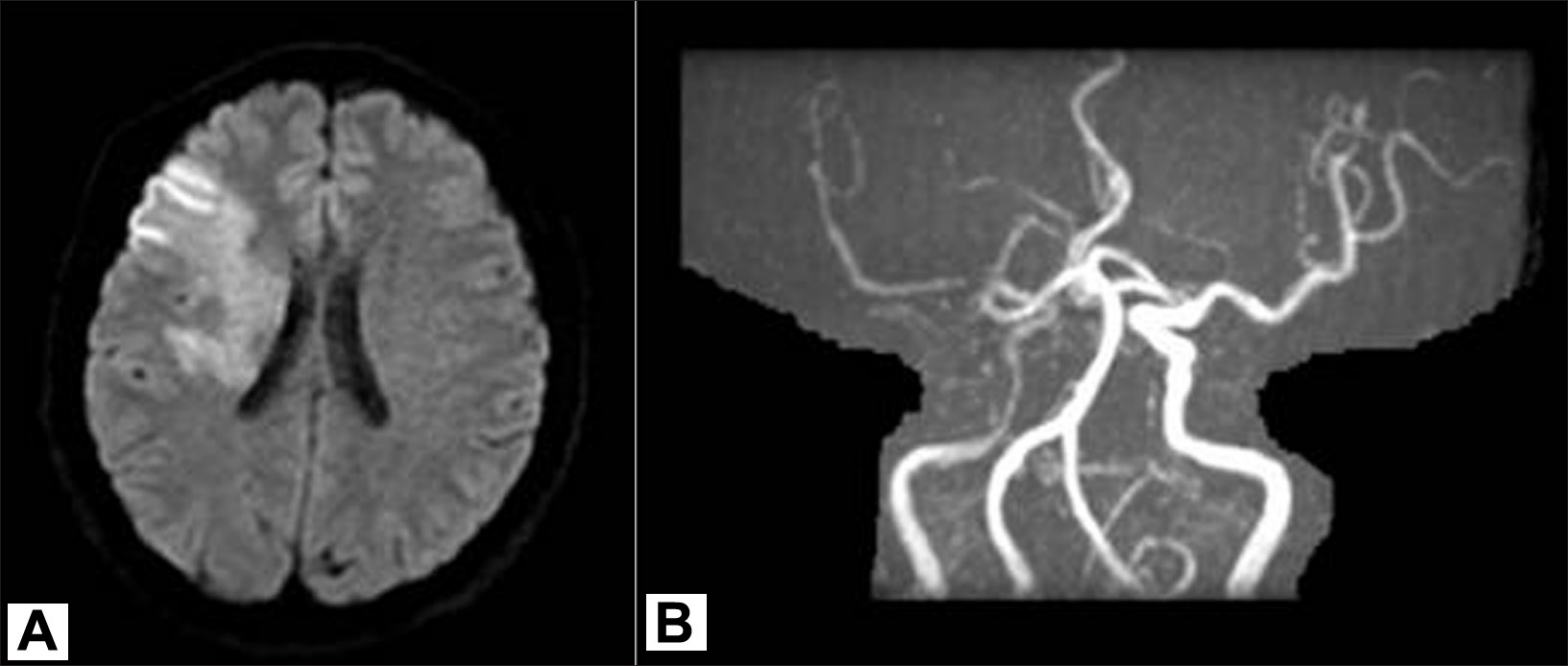
MRI and MRA scans showing acute infarction in the right frontotemporal area involving the right basal ganglia and corona radiata (A), and segmental narrowing of the siphon of the right ICA and the M1 segment of right MCA (B).

**Figure 2:**
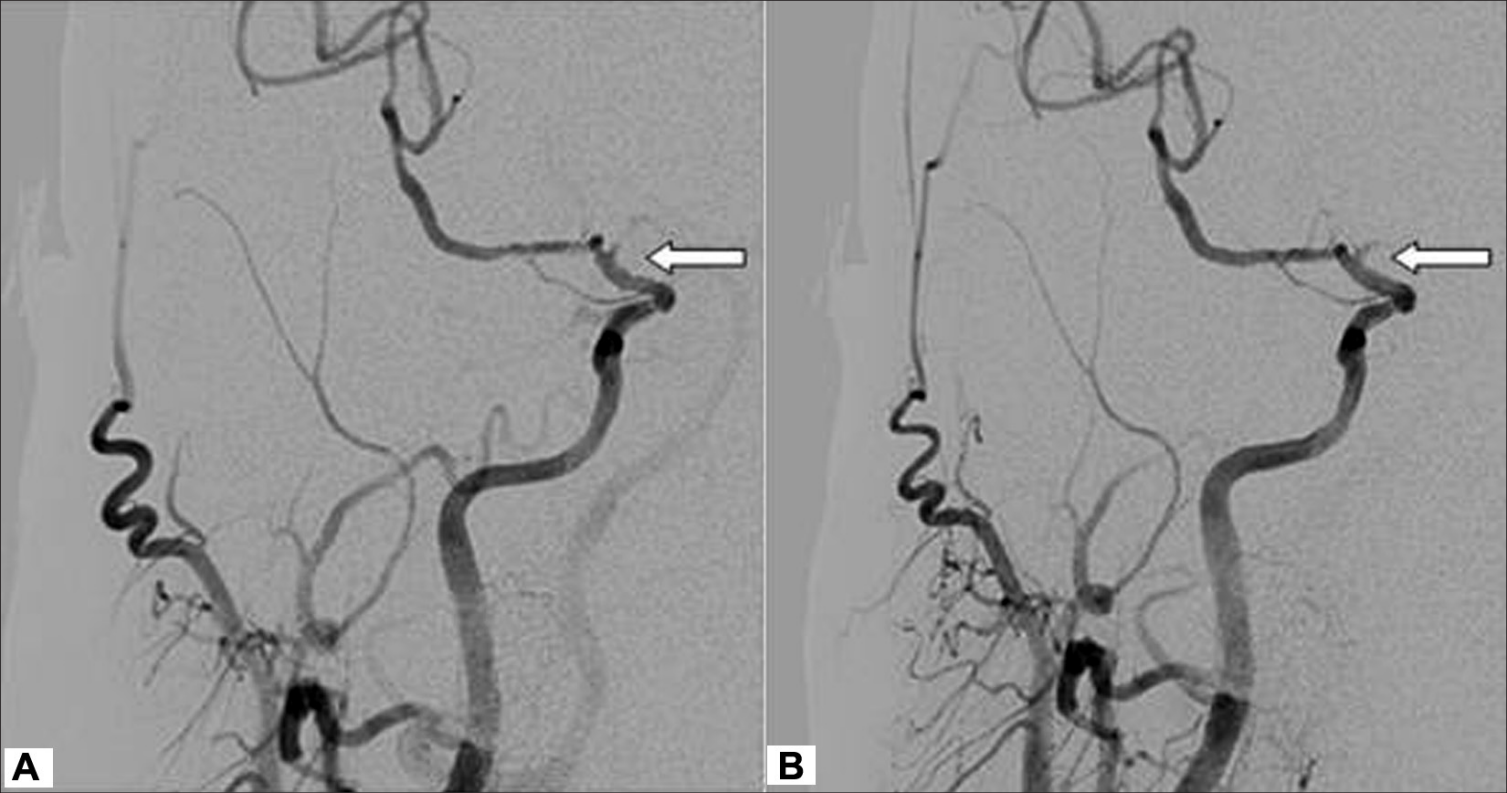
Narrowing and irregularity of the M1 lumen (A) was improved after treatment (B).

A 50-year-old man without any systemic disease or drug history was sent to a hospital after he incurred an electric shock injury while using a handicraft grinder by his two hands. This machine was adapted to a 60-Hz, 110-V alternating current supply. Upon receiving the shock, the man could not sever contact with the machine until 5 minutes later. He fell from a height of 1 meter on his buttocks. He remained conscious but experienced weakness and numbness in the left extremities, and his speech was slurred. He denied any head contusion happened during fall down. On arriving at the hospital, he was alert and his eyes were oriented to the right. Muscle strength on the left side was decreased, with a grade of 2 on the Medical Research Council Scale. The results of laboratory investigations, including complete blood count, coagulation function, and levels of electrolytes, serum glucose, liver enzymes and creatine kinase were all within the normal limits. The findings on chest X-ray scan, electrocardiogram and electrocardiogram were normal. A computed tomography (CT) scan of the brain showed no obvious abnormalities. With the impression of ischemic stroke with left hemiplegia, the patient was administered thrombolytic therapy with recombinant tissue plasminogen activator (r-tPA) within 3 hours after stroke onset, but no clinical improvement was noted. A magnetic resonance imaging scan of the brain showed an acute infarction in the right frontotemporal area involving the right basal ganglia and corona radiata. Magnetic resonance angiography showed segmental narrowing of the siphon of the right internal carotid artery (ICA) and the M1 segment of right middle cerebral artery (MCA) [Figure 1]. There were no stenosis or other abnormal finding founded in right common carotid artery or extracranial ICA by MRA study. Conventional angiography were arranged 4 hours after r-tPA, and showed a smaller-caliber lumen of the right ICA and irregular lumen of the distal M1 branch of the MCA. The M2 branch of the MCA proximal to the angular branch was patent. Focal narrowing of the M1 branch was manipulated by to-and-fro movement of the microcatheter, and vasospasm was treated by intravascular injection of nimodipine. After treatment, the flow in the right ICA region improved [Figure 2]. A rehabilitation program, including physical, occupational and speech therapy, was arranged for the patient. This patient can walk with quadricane for daily activities and dysarthria improved in 2 months.

## DISCUSSION

The weakness in the extremities experienced after electrical injury may be attributed to rhabdomyolysis, electrolyte imbalance, dehydration, keraunoparalysis, thermal injury, hypoxic encephalopathy, cerebral hypoperfusion, and mostly, vascular injury causing electrical coagulation, vasospasm, dissection, aneurysm formation and rupture. In this case, rhabdomyolysis, electrolyte imbalance and dehydration could be ruled out based on the clinical evaluation and laboratory results. Keraunoparalysis, which is transient paralysis of one or more extremities after lightning injury,[14] was less likely to be the cause of the weakness in our patient since in keraunoparalysis, the muscle strength and sensation usually return to normal within a few hours, which was not observed. Further, in this case, the conjugated eye deviation to the contralateral side of the hemiplegia implied a supratentorial lesion.

Janus *et al*, reported cases of 10 individuals who suffered from lightning injury with neurological complications, and only two of them had abnormal findings on cranial CT scans.[7] In our case, the acute ischemic stroke was better demonstrated on the MRI scan than the CT scan. Thus, MRI may not only be a better diagnostic tool for patients with acute neurological deficits after electric shock injury but also provide pathophysiological clues for further invasive treatment.

Thermal injury may be one of the mechanisms underlying cerebral damage.[12] The temperature of the cerebrospinal fluid may increase to as high as 145°F at 5 hours after electrocution. However, since the shortest current pathway did not pass through the brain in our patient, direct thermal injury is less likely to be the cause of ischemic stroke.

Acute stroke may result from vascular structural changes after electrical injury,[4] but acute infarctions or hemorrhage is not common after lightning accidents. Further, several cases of acute ischemic stroke after lightning showed watershed infarction due to hemodynamic alterations after cardiac arrest,[2] but a wedge-shaped infarction lesion on MRI was noted in only one report on lightning-induced cerebral infarction.[3] In our case, the wedge-shaped infarction in the right frontotemporal region was probably caused by territory occlusion due to vasospasm or embolism formation rather than systemic hypoperfusion and hypoxic encephalopathy.

A case of an 800-1500 V electrical injury followed by cerebral venous thrombosis.[10] In the same presentation in our case, there were no wounds founded during admission, and the electric injury should be considered as a macroshock. It is interesting to note that in case, the cerebral vessel injury lay outside the current pathway. In our case, the shortest current pathway was through the hands, arms, chest and heart. Even the electrocardiogram and echocardiogram showed normal findings or cardiac effects were most seen in microshock, transient arrhythmias during electric shock might not be completely excluded. Based on the MRI scan and angiographical findings, we assumed that the wedge-shaped infarction was probably induced by some small emboli that obstructed the narrowing vessel.

Both vasospasm and endothelial injury may contribute to vascular narrowing. Animal studies of electrical convulsive therapy showed segmental spastic constriction of the pial arteries and arterioles in the brain parenchyma, and the pial arteries were found to directly constrict with electrical stimulation.[6,9] Usually, these vasospasms persisted even after the stimulation was discontinued. Vascular endothelial damage may be related to electrical nonthermal effect, in addition to thermal effects. Lee *et al*,[8] used electroporation to describe this nonthermal effect, which causes structural changes in cell membranes and tissue damage by denaturation of intracellular proteins or changes in cell-membrane permeability.

Although acute ischemic stroke has not been observed in low-voltage electrical injuries, a long duration of contact may increase the probability of this occurrence. Stevenson reported that an alternating current with frequencies of 40-150 Hz would induce tetanic muscle contraction.[13] The “locking-on” phenomenon due to tetanic muscle contraction explained the persistent contact with the handicraft grinder in the case presented here. This is the reason why our patient could not avoid continued electrical injury, and mechanical injury due to prolonged tetanic muscle contraction may have been another cause of the acute ischemic stroke.

Post-lightning stroke patients may combine other neurological problems that should be close observation or management. Smith M.A.[11] reported one case with spinal cord myelin degeneration without inflammation after a low-voltage electrical injury. Post-lightning epilepsy, however, is a rare complication after electrical injury.[4] We should find if there existed other peripheral neuropathy, which usually combine with large area of burning injury.[11] Neuropsychological disorders are also important issues. Some neuropsychological and cognitive deficits resemble those of traumatic brain injury and post-traumatic stress disorder.[5] The etiologies and risk factors leading to the progression of neuropsychological disorders are not complete known.

## CONCLUSION

Electric shock injury with low-voltage alternating currents and a prolonged contact period may cause ischemic stroke. Vasospasms caused by the electrical injury may be the etiology of the stroke. MRI provides a better chance of early diagnosis and more clues for further investigation compared to a CT.
